# Editorial: Specific macroscopic brain changes in psychotic disorders

**DOI:** 10.3389/fnhum.2023.1141866

**Published:** 2023-02-06

**Authors:** Felix Brandl, Franziska Knolle, Chun Meng, Stefan Borgwardt

**Affiliations:** ^1^Department of Psychiatry and Psychotherapy, School of Medicine, Technical University of Munich, Munich, Germany; ^2^TUM-NIC Neuroimaging Center, School of Medicine, Technical University of Munich, Munich, Germany; ^3^Department of Neuroradiology, School of Medicine, Technical University of Munich, Munich, Germany; ^4^School of Life Science and Technology, University of Electronic Science and Technology of China, Chengdu, China; ^5^Department of Psychiatry, University of Lübeck, Lübeck, Germany

**Keywords:** psychotic disorders, schizophrenia, brain changes, specific, macroscopic

Psychotic disorders, covering mainly the schizophrenia spectrum and substance-induced psychosis, affect more than 1% of the population, are often chronic, and comprise debilitating symptoms like hallucinations, delusions, and cognitive impairments (American Psychiatric Association, 2013; McCutcheon et al., 2020). In recent years, many studies and meta-analyses have shown an association between psychotic disorders and macroscopic brain changes, particularly changes of brain structure, function, perfusion, and metabolism measured by magnetic resonance imaging (MRI) and positron emission tomography (PET) (McCutcheon et al., 2018; van Erp et al., 2018; Brandl et al., 2019; Gong et al., 2020; Sukumar et al., 2020). However, psychotic disorders overlap with other psychiatric disorders on the level of both symptoms and macroscopic brain changes (Goodkind et al., 2015; Kebets et al., 2019; Sha et al., 2019).

Therefore, we are yet to fully understand the macroscopic brain changes that are specific to psychotic disorders. Investigating changes specific to psychotic disorders may not only improve our pathophysiological and mechanistic understanding, but also—in the mid to long term—enable imaging-based differential diagnosis at early disease stages, and, thereby, informing the development of prognostic markers and specific treatment strategies. To achieve this, transdiagnostic approaches are necessary, for example comparisons of macroscopic brain changes in psychotic disorders, such as schizophrenia, with those in affective disorders, such as bipolar disorder or major depression (Brandl et al., 2019).

This Research Topic provides original studies and reviews examining macroscopic brain changes that are specific to psychotic disorders. The term “psychotic disorders” is understood as in DSM-5, covering mostly the schizophrenia spectrum and substance-induced psychosis (American Psychiatric Association, 2013). “Macroscopic brain changes” is understood as alterations of brain regions or systems in the millimeter/centimeter scale, which are detectable by *in-vivo* brain imaging. Finally, “specific” means that changes are more pronounced in psychotic disorders than in other psychiatric disorders, e.g., bipolar disorder.

Rootes-Murdy et al. compared gray matter alterations and symptom profiles of patients with schizophrenia and patients with bipolar I disorder, using a large structural MRI dataset. They showed that in general, patients with schizophrenia tend to have more severe symptom profiles and gray matter alterations, particularly in the temporal poles, than patients with bipolar I disorder. However, diagnostic boundaries were not clearly related to structural differences or distinct symptom profiles. They concluded that both disorders may track along an extensive spectrum of symptoms and brain correlates.

Schaub et al. started from observations that seasonal birth in winter and spring increases the risk of psychiatric disorders, for example schizophrenia and depression, possibly through pathological processes during neurodevelopment. They tested the effects of season of birth on gray matter volume (based on structural MRI) in a transdiagnostic sample of patients with schizophrenia and depression. They observed an effect of season of birth only in depression, but not in schizophrenia. Interestingly, and contrary to their expectations, hippocampal volume was lower in summer-born depressed individuals compared to winter-born individuals.

Takahashi et al. studied whether Heschl's gyrus duplication, a gyrification variant with increased prevalence in schizophrenia, is also present in bipolar disorder and major depression. Using structural MRI data, they showed a significantly higher prevalence of Heschl's gyrus duplication in patients with bipolar disorder, but not in patients with major depression, compared to healthy subjects. They concluded that the neurodevelopmental pathology of Heschl's gyrus duplication partly overlaps between schizophrenia and bipolar disorder, while its contribution in major depression appears distinct.

Cao et al. investigated the dyssynchrony of local brain activation in schizophrenia *via* a surface-based two-dimensional regional homogeneity approach, using resting-state fMRI data. They identified multiple aberrances both at the global and the local level, as well as links with illness duration and negative symptom severity. Some of their findings overlapped with abnormalities in bipolar disorder and depression, as reported in the literature. They concluded that the surface-based two-dimensional regional homogeneity approach could help with further exploring pathophysiological mechanisms of schizophrenia.

Rasmussen et al. examined associations between white matter microstructure and sleep-wake disturbances in individuals at ultra-high risk for psychosis. They used data from diffusion MRI, sleep questionnaire, and actigraphy. They observed an association between white matter microstructure of the corpus callosum and sleep disturbances in individuals at ultra-high risk for psychosis. They concluded that their findings suggest sleep disturbances as a potential treatment target.

Liang et al. investigated the link between structural MRI-based cortical thickness, MRS-based glutamate levels in the dorsal anterior cingulate cortex, and language dysfunction in patients with first-episode psychosis. Using a clustering approach, they identified a patient subgroup with widespread cortical thinning, higher glutamate levels in the dorsal anterior cingulate cortex, and reduced syntactic complexity and lexical cohesion. They concluded that their findings support the presence of detectable neurobiological subtypes of schizophrenia.

Salvador et al. applied a regularization approach (ridge regression), i.e., an innovative method to assess resting-state functional connectivity abnormalities, to resting-state fMRI data from patients with schizophrenia and healthy controls. They also compared their results to other measures of brain connectivity and dimensionality reduction. They observed widespread connectivity reductions in schizophrenia; the regularization approach outperformed the other methods. They concluded that regularization is a simple and sensitive alternative for quantifying functional brain connectivity.

In their Methods paper, Blair et al. propose a novel method for estimating complexity of brain activity, whose alteration in psychiatric disorders has already been shown by several studies. This method relies on dynamic functional connectivity to capture the distributed nature of brain activity and entropy measures to estimate global signal complexity. They applied their method to a sample of patients with obsessive-compulsive disorder, showing the robustness and consistency of this method compared with the existing literature.

In their Hypothesis and Theory paper, Liddle and Liddle review electrophysiological and fMRI-based evidence indicating imprecise predictive coding (i.e., the process of generating models of the world that are successively updated by sensory information) as a core pathological process in schizophrenia. They discuss macroscopic and molecular brain changes and their link with imprecise predictive coding and symptoms of schizophrenia, particularly disorganized and impoverished mental activity.

In conclusion, this Research Topic combines original studies and reviews concerning macroscopic brain changes in psychotic disorders, particularly compared to other psychiatric disorders such as bipolar disorder or major depression, and outlines promising areas for future research.

## Author contributions

All authors listed have made a substantial, direct, and intellectual contribution to the work and approved it for publication.
